# Changes of Motivational Variables in Patients with Multiple Sclerosis in an Exercise Intervention: Associations between Physical Performance and Motivational Determinants

**DOI:** 10.1155/2015/248193

**Published:** 2015-07-12

**Authors:** Wiebke Geertz, Anna-Sophie Dechow, Stefan Patra, Christoph Heesen, Stefan M. Gold, Karl-Heinz Schulz

**Affiliations:** ^1^Institute for Medical Psychology, University Medical Center Hamburg-Eppendorf, Martinistraße 52, Building W26, 20246 Hamburg, Germany; ^2^Competence Center for Sports and Exercise Medicine (Athleticum), University Medical Center Hamburg-Eppendorf, Martinistraße 52, 20246 Hamburg, Germany; ^3^Institute of Neuroimmunology and Multiple Sclerosis (INIMS), University Medical Center Hamburg-Eppendorf, Martinistraße 52, 20246 Hamburg, Germany; ^4^Department of Neurology, University Medical Center Hamburg-Eppendorf, Martinistraße 52, 20246 Hamburg, Germany

## Abstract

*Objectives*. This study examines the effects of a standardized fitness training on motivational factors such as the intention to be physically active, self-efficacy, perceived barriers, counterstrategies, and exercise specific social support in patients with progressive Multiple Sclerosis (MS) and the relation of these factors to physical performance. *Methods*. Moderately disabled patients with secondary or primary progressive MS (Expanded Disability Status Scale of 4–6) were randomized to a training group or a waitlist control group. Patients completed on average 20 sessions of training tailored to their individual fitness at baseline over a course of 8–12 weeks. Motivational variables (stage of change according to the transtheoretical model (TTM), self-efficacy, perceived barriers, counterstrategies, and exercise specific social support) were assessed via questionnaires at baseline and follow-up. *Results*. Forty patients completed the trial. We found significant effects on stages of change (*p* = .016) and self-efficacy (*p* = .014) and a trend in counterstrategies (*p* = .08). Significant correlations between change of physical performance during the exercise training and change in the TTM, perceived barriers, and counterstrategies were detected. *Conclusion*. This study indicates that tailored individual endurance training could stabilize self-efficacy and increase exercise motivation in patients with progressive MS. Motivational variables were related to the physical performance.

## 1. Introduction

Physical activity (PA) has been well documented as an important contributor to overall health and well-being [1–3]. The general level of physical activity, however, often is insufficient in the general population [4, 5]. Physical inactivity is associated with a higher risk for the development of chronic-degenerative diseases, cardiovascular-diseases, and diabetes [6, 7]. In particular in patients with chronic diseases as Multiple Sclerosis (MS), physical activity becomes more important. MS-patients suffer from impairments regarding their strength, coordination, and balance, as well as visual, cognitive, and affective deficits. As a result many MS-patients face progressive limitations of functioning in daily life [8]. There is substantial evidence in favor of regular exercise to be beneficial for patients with MS concerning strength, physical fitness, and mobility [9–11]. Although individuals agree upon the beneficial effects of physical activity on health, well-being, and quality of life [12, 13] most face motivational barriers which stop them from engaging in a more active lifestyle [14, 15]. Getting individuals with MS motivated to engage in physical activity may be particularly difficult due to disease-specific barriers such as fatigue and decreased mobility [16].

Research has identified several variables such as demographic factors, cognition, motivation, and social and physical environment that may influence levels of physical activity [17, 18] as well as self-initiated changes in health behavior [19].

A commonly used model to understand behavioral change for engaging in PA is the transtheoretical model (TTM) [20]. The TTM provides a framework for categorizing individuals' readiness to change their behavior as well as strategies for increasing PA and exercise behavior based on the individuals' motivation. Central construct of the TTM includes five discrete stages of change. Individuals move slowly over time through the stages (precontemplation, contemplation, preparation, action, and maintenance) of change that reflect behavioral intentions and the process from not considering engaging in a particular behavior to routine management. The model and its relationship to self-efficacy in health behaviors are generally supported by studies conducted with a variety of samples in the general population [21].

Plow et al. used the TTM model to assess PA among MS-patients [22]. They found the following distribution across the different stages: precontemplation (20%), contemplation (29%), preparation (14%), action (4%), and maintenance (31%). They further explored MS-related health problems, self-efficacy, and cognitive and behavioral processes of change in influencing stage of change. All of those sociocognitive variables significantly correlated with the stage of change.

Self-efficacy is needed to progress through the stages and engage in PA. It is described as a person's situation-specific belief in his or her capabilities to perform a behavior [19]. On the one hand a strong sense of self-efficacy facilitates motivation to engage in and maintain a physically active lifestyle. But on the other hand self-efficacy is also promoted by regular exercise [23]. In a review [24] of correlates of adults' participation in PA, a person's confidence in his or her ability to be physically active on a regular basis emerged as the most consistent correlate of PA behavior. Self-efficacy can increase through interventions and decrease after a period of inactivity [25]. Similar effects were shown by Motl and Gosney [26] in a meta-analysis in which a positive effect of exercise training interventions on individuals' self-efficacy in MS-patients was found. In addition to higher levels in self-efficacy, physically more active MS-patients also reported higher levels of social support (e.g., attachment, guidance, and reassurance of worth) [8].

Perceived barriers refer to individual's evaluation of the potential drawbacks and situations (e.g., limited time and that friends ask me out) that keep from engaging in PA. Barriers can be social (lack of social support), personal (lack of motivation and feeling lazy), and environmental (bad weather) [27]. Counterstrategies help managing perceived barriers.

The present study aims at examining motivational differences between MS-patients who are physically active in an exercise intervention compared to a waitlist control group that is not. We targeted sociocognitive variables that are known to be important to engage in PA: stages of change (TTM), perceived barriers, counterstrategies, self-efficacy, and social support. Furthermore we assessed the interrelation of the cognitive variables to the change in physical performance measured by the pre- and postspiroergometric assessments.

## 2. Methods

*(a) Study Design and Patient Recruitment*. Our data is part of a RCT in which exercise training (arm ergometry, rowing, and bicycle ergometry) was compared to a waitlist control group in progressive MS-patients with moderate disability. In our analysis we considered the training group as a whole and combined the participants of the different training groups. Beneficial effects of the exercise on aerobic fitness, depressive symptoms, fatigue, and cognitive functions were already reported by Briken et al. [9]. The training programs contained 16–24 standardized exercise sessions in 8–12 weeks. Participants took part in 2-3 sessions per week. The subjects in the waitlist control group were offered the intervention after three months. Patients were recruited through the MS outpatient clinic at the University Medical Center Hamburg-Eppendorf, as well as through advertisement on the website of the German MS society and leaflets left in neurologists' offices. The trial was approved by the ethics committee of the Chamber of Physicians, city of Hamburg, Germany (registration number PV 3689). Participants provided written informed consent prior to enrolment.

*(b) Inclusion and Exclusion Criteria*. All recruited patients had to meet diagnostic criteria for secondary or primary progressive MS and a moderate disability (Expanded Disability Status Scale (EDSS) of 4–6). Further inclusion criteria were age between 35 and 65 years and a maximum duration of the disease of 20 years. Patients were excluded if they had any medical contraindication for exercise therapy (cardiovascular or major orthopedic disease and general medical contraindications for increased aerobic activity).

*(c) Assessment Methods*. Subjects in both groups completed the following questionnaires prior to entering the study and after completion of the training or after the waiting period in the control group.

*Behavioral Change and PA outside the Study*. Participants' stage of change for PA was measured using the behavioral algorithm used by Basler et al. [28]. The questionnaire was used to evaluate participants' exercise behavior, reflecting the TTM stage of exercise. Participants were asked to choose which statement describes their present level of PA at best. The statements reflect the different stages of change (e.g., precontemplation: “No I am not physically active and I don't plan to start in the next 6 months” and maintenance: “I have been exercising for more than six months”). A second dichotomic item asks patients about their intentions in the last 6 months to be more physically active, for example, by buying sports equipment or choosing to walk instead of taking the car (0 = no and 1 = yes). Reliability and validity of the measure have been established [29]. In one further item we captured information about PA outside the study, asking the participants whether they were active and which kind of sports they carried out over the past 4 weeks.

*Self-Efficacy*. Self-efficacy for exercise, or the strength of the belief to be able to exercise, was evaluated by 3 items developed by Luszczynska and Schwarzer [30]. Participants rated on a 6-point Likert scale how confident they were from 1 (I am* not confident at all*) to 6 (I am* confident to 100%*) that they could engage in PA. Items differed regarding the self-efficacy to initiate, to maintain, and to restart PA. Within the present sample internal consistency for the scale in the study was *α* = .854.

*Social Support*. Social support was assessed using a 7-item scale [31]. Participants rated on a 4-point Likert scale (1 = never, 2 = sometimes, 3 = often, and 4 = always) how frequent friends and family, for example, helped plan activities around my exercise, encouraged me to start my exercise, offered to exercise with me, and encouraged me to stick to my exercise program. Within the present sample internal consistency for the scale in the study was *α* = .811.

*Perceived Barriers and Barrier Management*. Perceived barriers of engaging in PA were assessed using a 19-item questionnaire developed by Fuchs et al. [32]. Participants rated on a 4-point Likert scale how strong possible situational barriers keep them from exercising. Subjects had to report if and how strong they face the barriers which may impede the realization of exercising. Within the present sample internal consistency for the scale in the study was *α* = .812.

The patients' barrier management was measured with a dichotomous scale by Krämer and Fuchs [33] consisting of 15 items. Patients had to report whether they use specific strategies (e.g., I plan social activities involving exercise, I buy nice sporting clothes, I write the dates in my calendar, and I join an exercise group or class) or not (0 = I do not use this strategy and 1 = I use this strategy). Within the present sample internal consistency for the scale in the study was *α* = .813.

**Figure 1:**
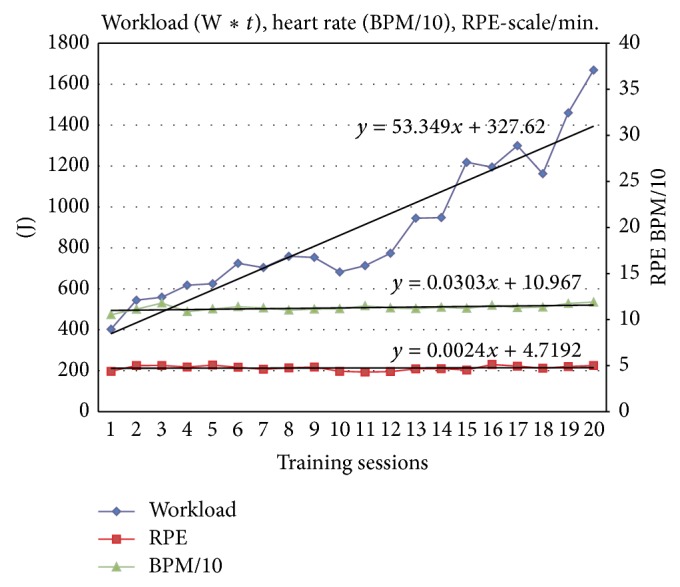
Changes in mean workload, heart rate, and ratings of perceived exertion in the training groups during 20 training sessions.* Note*. RPE: rating of perceived exertion, BPM: beats per minutes, and W: watt.

*Physical Performance*. We used the performance in watt measured in the spiroergometric assessment (before and after intervention) as an indicator of the physical capability. The difference between before and after intervention served as a marker of change in physical performance. The performance target was steadily increased with every training session whereas heart rate and subjective rating of exertion remained constant. An overview of the increased workload (changes in W*∗t*) throughout the training session is given in Figure 1.

*(d) Data Analysis*. Because of the small sample size and the difference in group size we chose a conservative approach. Differences between group and differences between changes in motivational variables (after minus before values) were tested using nonparametric independent samples Mann-Whitney *U* test. Spearman rank correlation coefficient was used to measure the associations between performance in watt and the motivational variables. Alpha was set to .05 for all tests of significance. To provide reliability of the questionnaires, internal consistency reliability was calculated using Cronbach's alpha coefficient. Due to missing data sample size varies. All analyses were performed using SPSS 18.0.

## 3. Results

### 3.1. Patient Sample and Demographic Data (Table 1)

80 patients showed interest in participating in the study. After the screening 47 patients met inclusion criteria and were randomized to one of the four treatment arms: ergometry, rowing, bicycle ergometry, and waitlist control. 40 (85%) patients, 10 in each group, finished the trial. Five patients did not complete the trial. Reasons for not completing the trial were logistic and mobility difficulties (*n* = 3), fatigue (*n* = 1), and an injury unrelated to the study (*n* = 1). Two patients did not attend to at least 16 training sessions and were excluded from calculations. No significant differences were found between subjects completing and dropping out of the study on any of the demographic characteristics (*p* ≤ .35). On average, patients attended to 20 training sessions (range: 16–24) (for more details see Briken et al., 2014).

### 3.2. TTM

**Figure 2:**
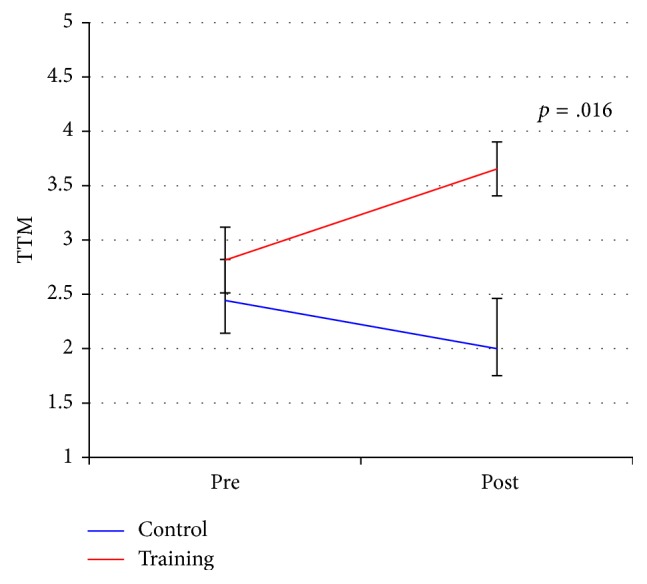
Effects of exercise training on stages of change for the training and control group.* Note*. Values as means; error bars indicate standard errors.

Before the start of the training the majority of patients (*N* = 26, 72%) reported being nonactive (precontemplation, contemplation stage, and preparation). 28% (*N* = 10) described themselves as active (action and maintenance stage). At baseline there was no significant difference between the two groups (Table 2). The training group progressed in stages of change due to exercise whereas the changes in the control group were in the opposite direction (*p* = .016; see Table 2 and Figure 2).

### 3.3. Physical Activity outside the Study

Prior to the study 13 out of 44 participants reported being active over the past 4 weeks (29%) (e.g., yoga, swimming, and fitness). At the second time point 24 out of 37 (64%) participants described themselves as being physically active, but only 7 participants reported PA (e.g., yoga, swimming, and fitness) other than partaking in the present study.

### 3.4. Self-Efficacy

**Figure 3:**
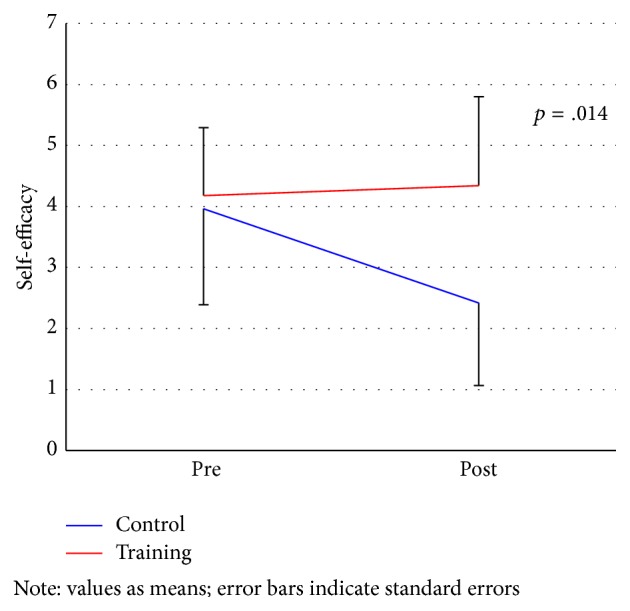
Change of self-efficacy in the training group and control group.* Note*. Values as means; error bars indicate standard errors.

While there was no significant difference between the two groups at baseline changes in self-efficacy differed significantly between the groups (*p* = .014; Figure 3; Table 2). This is due to a decrease in self-efficacy among waitlist group rather than an increase for the exercise group.

### 3.5. Perceived Barriers and Barrier Management

At baseline the top three barriers (named by more than 50%) which interfere “often/very often” with exercise participation were illness, being tired, and high financial costs. There was no difference between both groups (Table 2). After completion of the intervention fewer subjects in the exercise group stated “illness” (42%) as a strong barrier. In the control group “illness” (75%) remained a strong barrier.

Prior to the training the difference of used strategies to overcome situational barriers between the two groups is not significant (Table 2). The alteration of used counterstrategies differs by trend (*p* = .08; Table 2). Participants of the training group reported a stable use of counterstrategies, whereas subjects in the control group used fewer counterstrategies over time.

### 3.6. Social Support

At baseline, the two groups did not differ significantly in their reports on exercise specific social support (Table 2). After the exercise program, participants in the training groups reported slightly higher levels of social support, whereas participants in the control group reported decreased levels of social support. These different changes however are not statistically significant (Table 2).

### 3.7. Associations between Physical Performance and Motivational Variables

We saw significant moderate correlations between changes of an increase in performance during exercise (change watt) and the change of stages in the TTM model (*r*_*s*_(23) = .553, *p* = .006), the changes of perceived barriers (*r*_*s*_(23) = −.431, *p* = .04), and the change in use of counterstrategies (*r*_*s*_(17) = −.533, *p* = .028) (Table 3).

## 4. Discussion

The study examined stage of change, self-efficacy, and social support for a better understanding of determinants that encourage engaging in physical exercise in progressive MS-patients. It provides evidence for effects of exercise on changes in motivational variables. During the weeks of training higher stages in the TTM, a stabilizing effect on self-efficacy, a change in the perception of barriers, and a stabilization of coping strategies were detected.

In our sample, most of the patients (72% in TTM; 71% in the item asking for PA outside the study) were inactive prior to the intervention. This matches research that indicates very low levels of PA in MS-patients [22, 34, 35]. As expected after 8–12 weeks of training the participants reported a significantly higher stage in the TTM framework compared to the control group. The association between change in TTM stages and change in physical performance provides support of the validity of the algorithm.

Our results indicate that the level of self-efficacy of exercising patients remains stable over the period of training compared to the decreasing level of self-efficacy in the physically nonactive patients. This result does not completely match the finding from McAuley and Blissmer [25] who derived a higher level of self-efficacy after an exercise intervention but also showed a lower level of self-efficacy after a period of physical inactivity. A possible explanation for the missing increase is the already high level of self-efficacy in our groups at baseline. We assume that this is due to the fact that primarily patients with a higher level of self-efficacy take part in an exercise based intervention. On the one hand the decreasing level of the waitlist group is unexpected as one would assume that their level of self-efficacy would rise given that they soon start to exercise and have high expectations about being active but on the other hand the waiting period is probably too long to keep those expectations at a higher level.

At baseline, more than 50% of all patients reported tiredness, illness, and high financial costs as restricting factors. This finding is consistent with Netz et al. [36] who detected in a population-based study illness, high costs, and negative feelings as strong barriers to PA. In contrast to our results they also identified lack of time, lack of energy, and lack of motivation as strong barriers. It seems that MS-patients compared to the healthy population report more frequently factors related to their disease as perceived strong barriers. After completion of the 8–12 weeks of training the perception of the illness as a barrier differs between the intervention and the control group. This result is supported by the negative correlation between change in watt and change in perceived barriers in the intervention group. It seems that a period of active training contributes to a change in the perception of barriers in a positive, less restricting way. One possibility for the explanation of this difference is that disease related barriers do not represent objective limitations due to the illness but rather anxieties and expectations. After being physically active, participants possibly notice that their prior expectations were not confirmed and that their worries were not reasonable. High costs as a perceived barrier as reported by more than 50% of our subjects represent a general public health problem. Although evidence of health related benefits of regular exercise accumulates, health insurances do not regularly cover such costs.

Our data also show a positive trend in counterstrategies. The subjects of the intervention group reported stable levels of barrier management. In contrast, participants in the control group used fewer counterstrategies at the second assessment. This finding seems counterintuitive, as one would expect an increase in the use of counterstrategies after a period of exercise. However, considering the lower level of perceived barriers after the intervention period it seems as if because of the reduction of former barriers fewer counterstrategies are needed for being active.

At baseline, the perception of exercise specific social support was on a similar level in both groups. After completion of the training, the training group reported a slightly though not significant higher level of social support. This could be explained by the enhanced awareness of the social environment, regular attention from therapists, social support from other patients, and probably the need of social support in order to partake in all training sessions. Results are in line with Motl et al. [8], who found higher levels of social support in physically active MS-patients.

Overall, the study is consistent with Plow et al. [22] and underscores the association between physical activity and several motivational factors by substantial correlations of these variables. However the correlations preclude inferences about the causal relationship. Whether a higher level of PA improves self-efficacy and social support and decreases perceived barriers or a higher level of these factors enhances PA is debatable. Most likely there is a constant interaction between self-efficacy, barriers, counterstrategies, and PA. Patients feel more encouraged to maintain PA after recognizing improvements in physical performance and benefits in daily living. This in turn further improves PA and motivation. However, this interaction most likely is also working towards decreasing PA and motivation.

The strength of the current study is the individually tailored intervention. Physical capabilities vary between the patients. Since the strongest barriers are illness-related, it is important to ensure the patients to feel safe and being able to exercise. This need is guaranteed in individually tailored and supervised exercise programs.

There are several limitations of this study, which have to be considered. First, this trial comprised a small sample and difference in group size thus limiting statistical power to detect differential effects, for example, between the different subgroups of the intervention. Second, we need to be aware of the fact that the waitlist control group received less frequent attention by the therapists. Therefore, answers in the self-report measures such as the questionnaires to social support, barriers, and self-efficacy may have been affected by nonspecific factors, such as attention from the therapist or social support from other patients. Finally, the patients in this study may not be representative of persons with MS in general as we only included patients with secondary or primary progressive MS. Furthermore, due to the advertisements as an exercise based study, there might have been an upward bias towards participants with increased self-efficacy and motivation compared to the general population and to the average patients with MS.

The findings require replication in larger samples and longitudinal studies. Taken together, this study underscores the importance of further examining modifiable variables that correlate with PA. Given the data from this and other studies, future studies should examine further mechanisms that explain the inactivity of MS-patients as well as the long-term effects of exercise on sociocognitive and motivational factors. This will help to increase the understanding of underlying mechanisms for the low level of PA among MS-patients. Furthermore, it will help to develop empirically supported exercise intervention programs that manage to maintain PA among MS-patients and in turn improve MS-patients' quality of life. In comparison to interventions designed for the general population, exercise based interventions for persons with MS also need to address disease related anxieties.

## 5. Conclusion

We detected a low level of PA among our participants. Considering the vital benefits of PA for MS-patients this finding is of utmost relevance. Prior to the intervention the main barriers for our patients were disease related. Those disease related barriers were not perceived as strong barriers after completion of the training period. Our results show that MS-patients benefit from participation in tailored individual endurance training. Short term professionally guided exercise could be an access to a more active lifestyle.

## Figures and Tables

**Table 1:** Clinical baseline characteristics of training and control group.

	Group	
	Training	Control
*N*	30	10
Age	49.73 ± 8.013	50.4 ± 7.575
Sex (m/f)	18/12	4/6
EDSS	4.867 ± 0.8087	4.85 ± 0.8515
MS type (SPMS/PPMS)	22/8	8/2
Drop-outs (*n*, %)	6 (20%)	1 (10%)
Disease duration (years)	18.9 ± 6.286	15.07 ± 9.848

*Note*. EDSS: Expanded Disability Status Scale. PPMS: Primary Progressive Multiple Sclerosis. SPMS: Secondary Progressive Multiple Sclerosis. Data given as mean ± standard deviation.

**Table 2:** Means (M), standard deviations (SD), and median (Mdn.) of motivational variables of the groups for pre- and posttreatment assessments.

	*n*	Training			*n*	Control			*p* value at baseline	*p* value of response
		M	SD	Mdn.		M	SD	Mdn.		
TTM pre.	27	2.82	1.57	2.00	9	2.44	1.13	2.00	.815	.016
TTM post.	26	3.65	1.26	4.00	8	2.00	1.31	2.00		
Self-efficacy pre.	29	4.18	1.11	4.30	8	3.96	1.58	4.33	.959	.014
Self-efficacy post.	25	4.34	1.46	4.70	8	2.42	1.36	2.00		
Barriers pre.	27	1.91	0.48	1.80	8	2.00	0.78	2.15	.665	.570
Barriers post.	26	1.69	0.35	1.70	8	2.03	0.53	1.98		
Counterstrategies pre.	22	0.61	0.24	0.65	8	0.59	0.25	0.55	.653	.080
Counterstrategies post.	23	0.59	0.27	0.50	8	0.38	0.21	0.35		
Social support pre.	28	2.16	0.78	2.00	8	2.05	0.56	2.05	.818	.155
Social support post.	25	2.43	0.76	2.60	8	1.79	0.65	1.75		

*Note*. TTM: transtheoretical model, significant *p* values (*p* < .05). *p*: Mann-Whitney *U* test.

**Table 3:** Correlations of the changes in motivational variables and the change in performance in the exercise sessions for participants of the training group.

	TTM change	SE change	B change	CS change	SS change
TTM change	—				
SE change	0.143	—			
B change	−0.101	−0.397	—		
CS change	0.071	0.198	−0.058	—	
SS change	**0.524**	−0.127	0.028	0.155	—
Watt change	**0.553**	0.309	**−0.431**	**−0.533**	−0.02

*Note*. Significant correlations (*p* < .05) are in boldface; TTM: transtheoretical model, SE: self-efficacy, B: barriers, CS: counterstrategies, and SS: social support.
